# Immunogenicity of COVID-19 mRNA vaccines in immunocompromised patients: a systematic review and meta-analysis

**DOI:** 10.1186/s40001-022-00648-5

**Published:** 2022-02-12

**Authors:** Mohammad-Mehdi Mehrabi Nejad, Fatemeh Moosaie, Hojat Dehghanbanadaki, Abdolkarim Haji Ghadery, Mahya Shabani, Mohammadreza Tabary, Armin Aryannejad, SeyedAhmad SeyedAlinaghi, Nima Rezaei

**Affiliations:** 1 Department of Radiology, School of Medicine, Advanced Diagnostic and Interventional Radiology Research Center (ADIR), Imam Khomeini Hospital, Tehran University of Medical Sciences (TUMS), Tehran, Iran; 2 School of Medicine, Tehran University of Medical Sciences, Tehran, Iran; 3 Diabetes Research Center, Endocrinology and Metabolism Clinical Sciences Institute, Tehran University of Medical Sciences, Tehran, Iran; 4 Division of Pulmonary, Allergy and Critical Care Medicine, Department of Medicine, University of Pittsburgh, Pittsburgh, PA USA; 5 Experimental Medicine Research Center, Tehran University of Medical Sciences, Tehran, Iran; 6 Iranian Research Center for HIV/AIDS, Iranian Institute for Reduction of High-Risk Behaviors, Tehran University of Medical Sciences, Tehran, Iran; 7 Research Center for Immunodeficiencies, Children’s Medical Center, Tehran University of Medical Sciences, Tehran, Iran; 8 Department of Immunology, School of Medicine, Tehran University of Medical Sciences, Qarib St, Keshavarz Blvd, 1419733141 Tehran, Iran

**Keywords:** COVID-19, SARS-CoV-2, Vaccination, Immunocompromised patient, Malignancy, Transplantation, Autoimmune, Efficacy

## Abstract

**Background:**

Immunocompromised (IC) patients are at higher risk of severe SARS-CoV-2 infection, morbidity, and mortality compared to the general population. They should be prioritized for primary prevention through vaccination. This study aimed to evaluate the efficacy of COVID-19 mRNA vaccines in IC patients through a systematic review and meta-analysis approach.

**Method:**

PubMed-MEDLINE, Scopus, and Web of Science were searched for original articles reporting the immunogenicity of two doses of mRNA COVID-19 vaccines in adult patients with IC condition between June 1, 2020 and September 1, 2021. Meta-analysis was performed using either random or fixed effect according to the heterogeneity of the studies. Subgroup analysis was performed to identify potential sources of heterogeneity.

**Results:**

A total of 26 studies on 3207 IC patients and 1726 healthy individuals were included. The risk of seroconversion in IC patients was 48% lower than those in controls (RR = 0.52 [0.42, 0.65]). IC patients with autoimmune conditions were 54%, and patients with malignancy were 42% more likely to have positive seroconversion than transplant recipients (*P* < 0.01). Subgroup meta-analysis based on the type of malignancy, revealed significantly higher proportion of positive seroconversion in solid organ compared to hematologic malignancies (RR = 0.88 [0.85, 0.92] vs. 0.61 [0.44, 0.86], *P* = 0.03). Subgroup meta-analysis based on type of transplantation (kidney vs. others) showed no statistically significant between-group difference of seroconversion (*P* = 0.55).

**Conclusions:**

IC patients, especially transplant recipients, developed lower immunogenicity with two-dose of COVID-19 mRNA vaccines. Among patients with IC, those with autoimmune conditions and solid organ malignancies are mostly benefited from COVID-19 vaccination. Findings from this meta-analysis could aid healthcare policymakers in making decisions regarding the importance of the booster dose or more strict personal protections in the IC patients.

**Supplementary Information:**

The online version contains supplementary material available at 10.1186/s40001-022-00648-5.

## Introduction

Immunocompromised (IC) patients include individuals with over-activation or suppression of the immune system due to primary disease or treatment regimens [1]. The most common conditions in this group are malignancies, inherited or acquired immunodeficiency diseases, autoimmune diseases, transplant recipients, and other conditions requiring long-term corticosteroid [1]. IC conditions are estimated to affect approximately 2.7% of United States adults [2]. Such patients are at higher risk of severe SARS-CoV-2 infection, extended hospitalization, intensive care admission, and mortality compared to the general population [3–7]. Besides, prolonged viral shedding and potential sources of novel SARS-CoV-2 variants in this population are also of particular importance [8–10]. Thus, IC patients should be prioritized for primary prevention through Coronavirus infectious disease 2019 (COVID-19) vaccination.

Global efforts have been taken to develop SARS-CoV-2 vaccines since the initiation of the current COVID-19 pandemic. The mRNA vaccines (i.e., mRNA-1273 and BNT162b2) are the most commonly approved vaccines worldwide which are utilized in different clinical trials on a global scale [11]. The overall efficacy and safety of COVID-19 vaccines in phase III trials were promising [12], sparking global hope toward ending the current outbreak. However, the application of COVID-19 vaccines in patients with impaired immune systems remains an ongoing subject of debate as they were excluded from the original trials [13, 14]. Due to either the primary disease or the immunosuppressive treatments, IC patients are more likely to show a weak or suboptimal immune response to COVID-19 vaccines, given previous studies on influenza vaccines [15]. Hence, real-world statistics regarding the efficacy of COVID-19 vaccines are required to provide physicians a better insight towards decision-making in this group of high-risk patients.

This study aimed to systematically review the literature and analyze the pooled effectiveness of COVID-19 vaccination in IC patients compared to healthy controls using meta-analysis. We also assessed the efficacy of mRNA vaccines in IC patients based on their etiological factors, including malignancy, transplantation, and autoimmune diseases.

## Methods and materials

### Protocol and literature search

This systematic review and meta-analysis study was conducted according to the Preferred Reporting Items for Systematic Reviews and Meta-Analyses (PRISMA) guidelines.

PubMed-MEDLINE, Scopus, and Web of Science were searched for original articles reporting the efficacy in adult patients with IC conditions between June 1, 2020 and September 1, 2021. The search terms were as follows: ((COVID-19) OR (SARS-CoV-2) OR (novel coronavirus)) AND ((vaccine) OR (vaccination)) OR (vaccinated)) AND ((immunocompromised) OR (immunosuppressed) OR (corticosteroid) OR (chemotherapy) OR (cancer) OR (malignancy) OR (rheumatologic disease) OR (immunodeficiency) OR (autoimmune) OR (AIDS) OR (HIV) OR (transplant)).

The references of the selected articles were further screened to search for potentially relevant articles. Two reviewers independently performed the literature search, and any disagreement regarding study inclusion was resolved by consensus. The authors were not blinded to the authors, institutions, or journals while selecting studies or extracting data. EndNote version ×9 was used for literature management.

### Eligibility criteria

Studies investigating the immunogenicity of COVID-19 mRNA vaccination in IC patients were eligible for inclusion. The included studies met the following criteria. (1) Population: studies on IC patients with a sample size ≥ 30 participants and control group of healthy individuals. IC patients included patients with solid organ or hematologic malignancies who receive chemotherapy, patients with inherited or acquired immunodeficiency diseases, patients with autoimmune or rheumatologic diseases, patients with other conditions (i.e., asthma) receiving long-term corticosteroid, and transplant recipients. (2) Intervention: mRNA COVID-19 vaccination. (3) Study design: all retrospective and prospective studies, as well as clinical trials with a healthy control group, were included. (4) Outcomes: the main outcome of this study was seroconversion in IC patients using anti-SARS-CoV-2 spike IgG after the second dose of COVID-19 mRNA vaccines. The subgroup analysis was performed to determine the efficacy of COVID-19 mRNA vaccines in different groups of patients based on the etiology of the IC condition.

The exclusion criteria were as follows: (1) reviews and editorials; (2) case reports or case series < 30 patients; (3) partially overlapping patient cohorts; (4) articles not written in English; (5) single-arm studies or with a non-healthy control group; and (6) non-human studies. Two reviewers independently reviewed the literature in consensus.

### Data collection

Eligible studies were evaluated by two experts independently. The following data were extracted from each included publication: author, date of publications, country of origin, study design, study sample size, the definition of IC conditions, inclusion and exclusion criteria, the number of IC patients, variables matched, the proportion of male, mean age, duration of disease, type and etiology of the immunodeficiency and its proportion to the total population, type of vaccination, and efficacy of the vaccination.

Any conflicts in data extraction were discussed or consulted by a third expert and resolved.

### Quality assessment

National Institutes of Health (NIH) quality assessment tool [16] was used to evaluate the included studies. The scores of 11–14, 6–10, and 0–5 were considered good, fair, and poor quality, respectively. Moreover, the studies were evaluated in terms of methodology by two experts independently; any conflict of opinion was discussed or referred to a third expert and resolved.

### Statistical analyses

STATA version 16 for Windows (Stata Corp, College Station, Texas) was utilized for the meta-analysis. At least three studies in each group were required to synthesize the data on outcomes. The heterogeneity of studies was measured using I^2^ or Q test. A fixed model was employed if the heterogeneity of studies was below 40% and a random effect model in case of heterogeneity above 40%. Effect measures were calculated as relative risk (RR), but relative risk reduction (RRR; 1-RR) values were reported in the results section for better interpretation. Also, based on the heterogeneity of studies, either meta-regression analysis or subgroup analysis was performed for potential moderators. Moreover, funnel plot asymmetry and the Eggers test were used to assess publication bias. In case of significant publication bias, the adjustment was performed for the effect size using the trim-and-fill method. A *P*-value less than 0.05 was considered statistically significant.

## Results

### Study selection

**Fig. 1:**
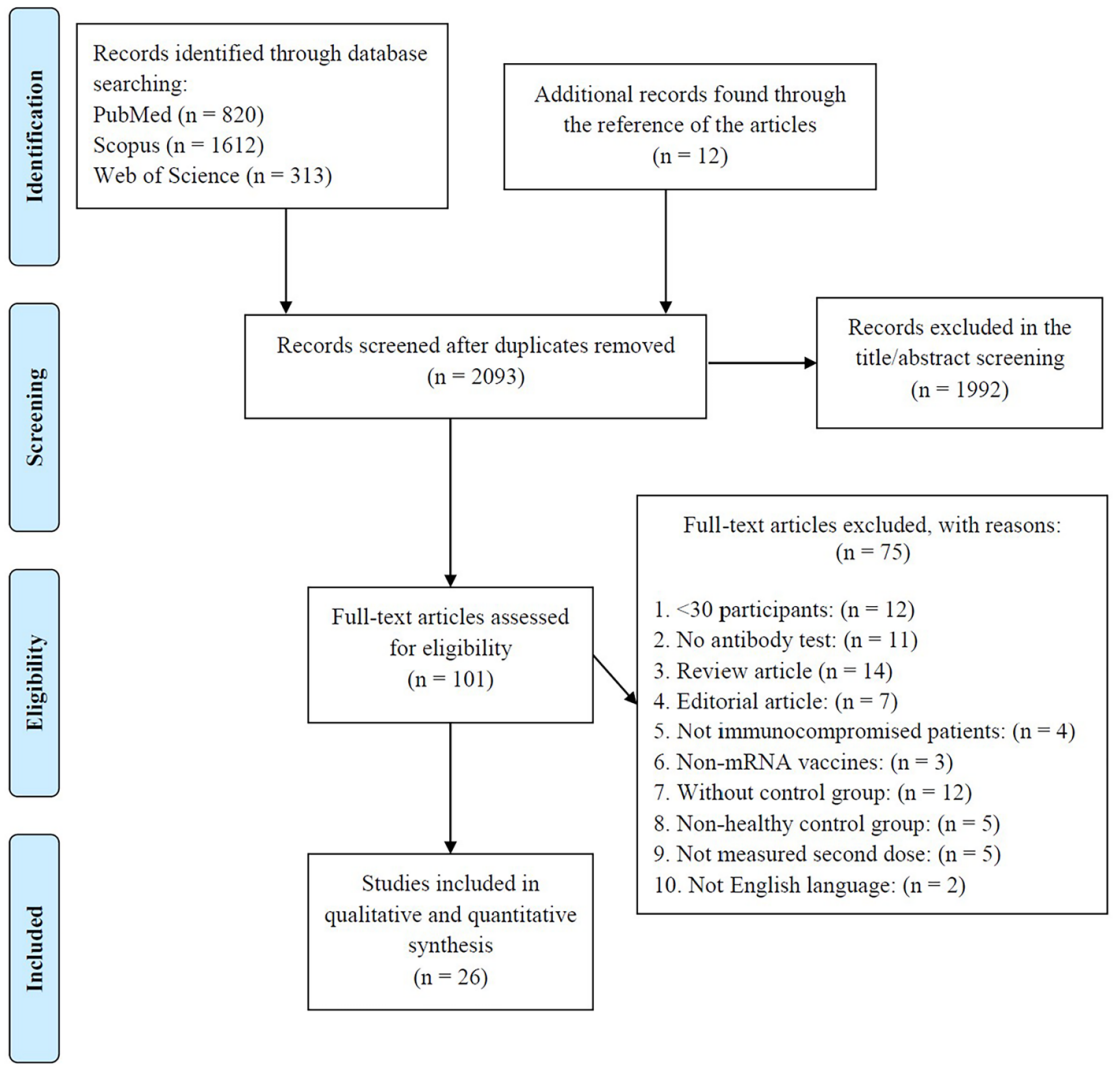
The PRISMA 2009 flow diagram of the study

The study selection flowchart is presented in Fig. 1. The literature search, after removing duplicates, resulted in 2093 studies, of which 1992 were considered irrelevant following title and abstract screening. Of the remaining 101, a further 75 were removed according to the exclusion criteria. Therefore, in total, 26 studies [17–42] were eligible for the meta-analysis of seroconversion after the second dose of the vaccine.

### Characteristics of included studies

Characteristics of the included studies are provided in Table 1. All 26 included studies on 3207 IC patients and 1726 healthy controls showed that 65.8% IC patients and 99.2% healthy controls had seropositive IgG tests following the second dose of COVID-19 mRNA vaccines. All of the studies were conducted in 2021. Sample sizes, from which relevant data were available for extraction, varied from 40 to 807. Participants’ mean age ranged from 42 to 71.4 years. The majority of the studies [19, 21, 24–26, 28, 30–35, 37–42] had a prospective cohort design (*n* = 18). Five studies [18, 20, 22, 23, 29] had a retrospective cohort design and three [17, 27, 36] were cross-sectional.

**Table 1:** Details of the data presented by the included studies

Study (first author)	Country	Study design	Total sample size	Case			Control			Etiology of IC condition	Type of vaccine
				No. of cases	Male, % of cases	Age	No. of non-cases (if applicable)	Male, % of non-cases	Age		
Sattler A	Germany	Prospective cohort	78	39	71.8	57.3	39	51.2	53.0	Transplant	BNT162b2 (Pfizer/BionTech)
Rincon-Arevalo H	Germany	Prospective cohort	75	40	70	62.4 [51.2–69.5]*	35	57.1	51 [34–80]*	Transplant	BNT162b2 (Pfizer/BionTech)
Korth J	Germany	Prospective cohort	46	23	48	57.7	23	39	44.4	Transplant	BNT162b2 (Pfizer/BionTech)
Rabinowich L	Israel	Cross-sectional	105	80	70	60.1	25	32	52.7	Transplant	BNT162b2 (Pfizer/BionTech)
Schramm R	Germany	Prospective cohort	100	50	64	55	50	34	47	Transplant	BNT162b2 (Pfizer/BionTech)
Cao J	USA	Retrospective cohort	47	37	72.9	64 [50–69]*	10	20	66 [57–75]*	Transplant	mRNA-1273 (Moderna) or BNT162b2 (Pfizer/BionTech)
Grupper A	Israel	Retrospective cohort	151	136	81.7	58.6	25	32	52.7	Transplant	BNT162b2 (Pfizer/BionTech)
Marinaki S	Greece	Prospective cohort	150	34	79.4	60 [49.1–68.4]*	116	–	–	Transplant	BNT162b2 (Pfizer/BionTech)
Rashidi-Alavijeh J	Germany	Prospective cohort	63	43	60.5	57 [49–64]*	20	45	43.5 [38–53.5]*	Transplant	BNT162b2 (Pfizer/BionTech)
Hod T	Israel	Prospective cohort	322	120	80	59.7	141	30.2	57.04	Transplant	BNT162b2 (Pfizer/BionTech)
Stumpf J	Germany	Prospective cohort	512	368	65.5	57.3	144	23.6	48	Transplant	(a) mRNA-1273 (Moderna) (*n* = 143); (b) BNT162b2 (Pfizer/BionTech) (*n* = 369)
Firket L	USA	Retrospective cohort	40	20	45	51.2	20	65	48.3	Transplant	BNT162b2 (Pfizer/BionTech)
Peled Y	Israel	Prospective cohort	213	77	64	62 [49–68]*	136	37	63	Transplant	BNT162b2 (Pfizer/BionTech)
Monin L	UK	Prospective cohort	205	151	52	73 [64.5–79.5]*	54	52	40.5 [31.3–50]*	Malignancy	BNT162b2 (Pfizer/BionTech)
Pimpinelli F	Italy	Prospective cohort	128	92	53/2	70*	36	0	81	Malignancy	BNT162b2 (Pfizer/BionTech)
Massarweh A	Israel	Prospective cohort	180	102	57	66 [56–72]*	78	32	62 [49–70]*	Malignancy	BNT162b2 (Pfizer/BionTech)
Agbarya A	Israel	Cross-sectional	355	140	54	65.3	215	37.2	62.5	Malignancy	BNT162b2 (Pfizer/BionTech)
Herishanu Y	Israel	Prospective cohort	219	167	67.1	71 [63–76]*	52	–	69 [63–73.7]*	Malignancy	BNT162b2 (Pfizer/BionTech)
Iacono D	Italy	Cross-sectional	108	36	41.6	82*	72	–	≥ 66	Malignancy	BNT162b2 (Pfizer/BionTech)
Malard F	France	Retrospective cohort	225	195	60	68.9*	30	–	–	Malignancy	BNT162b2 (Pfizer/BionTech)
Eliakim-Raz N	Israel	Prospective cohort	161	95	58	65 [56–72]*	66	32	62 [50–70]*	Malignancy	BNT162b2 (Pfizer/BionTech)
Herzog Tzarfati K	Israel	Prospective cohort	423	315	56	71 [61–78]*	108	44	69 [58–74]*	Malignancy	BNT162b2 (Pfizer/BionTech)
Reuken P	Germany	Prospective cohort	55	28	46.4	42 [36–59]*	27	–	–	Autoimmune	BNT162b2 (Pfizer/BionTech)
Geisen UM	Germany	Retrospective cohort	68	42	35.7	50.5	26	30.8	37.5	Autoimmune	mRNA-1273 (Moderna) or BNT162b2 (Pfizer/BionTech)
Furer V	Israel	Prospective cohort	807	686	30.7	59 [19–88]*	121	35	50*	Autoimmune	BNT162b2 (Pfizer/BionTech)
Prendecki M	UK	Prospective cohort	155	85	52.1	52 [39.9–63.9]*	70	–	41.4*	Autoimmune	BNT162b2 (Pfizer/BionTech)

^*^Median [IQR] is reported; otherwise the mean is reported

### Seroconversion in immunocompromised patients vs. controls

**Fig. 2:**
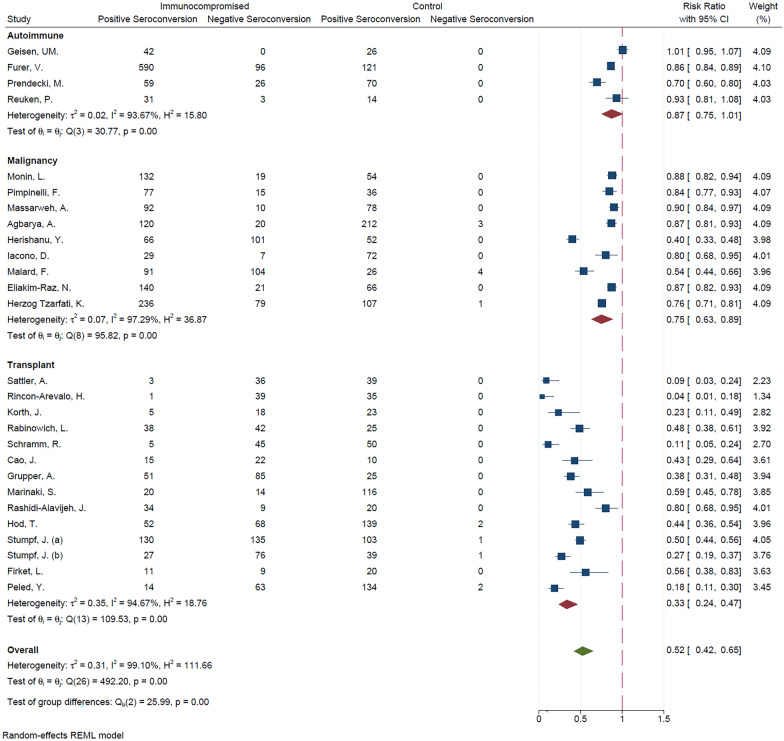
Meta-analysis of seroconversion in immunocompromised patients vs controls, based on type of immunodeficiency

Meta-analysis of 26 studies (I^2^ = 99.10%) revealed that positive seroconversion risk in IC patients were 48% lower than healthy controls. (RRR = 0.48; 95% CI 0.35, 0.58; *P* < 0.01). Subgroup meta-analysis based on the type of IC (i.e., autoimmune, transplant, and malignancy), revealed a statistically significant between-group difference (*P* < 0.01) (Fig. 2). When comparing each two subtypes of immunodeficiency, the results showed that IC patients due to transplant were less likely to develop positive seroconversion than IC patients due to autoimmune disorder (*P* < 0.01) as well as IC patients due to malignancy (*P* < 0.01). There was no statistically significant difference in seroconversion between IC patients with an autoimmune disorder and those with malignancy (*P* = 0.19).

### Seroconversion in patients with autoimmune disease vs. controls

Four [21, 22, 35, 38] of the included studies were conducted on IC patients with autoimmune immunodeficiency. Although the proportion of positive seroconversion in these patients was lower than the controls, the pooled analysis showed no statistically significant difference in relative risk reduction of seroconversion between two groups (RRR = 0.13; 95% CI − 0.01, 0.25; *P* = 0.07) (Fig. 2).

### Seroconversion in patients with malignancy vs. controls

**Fig. 3:**
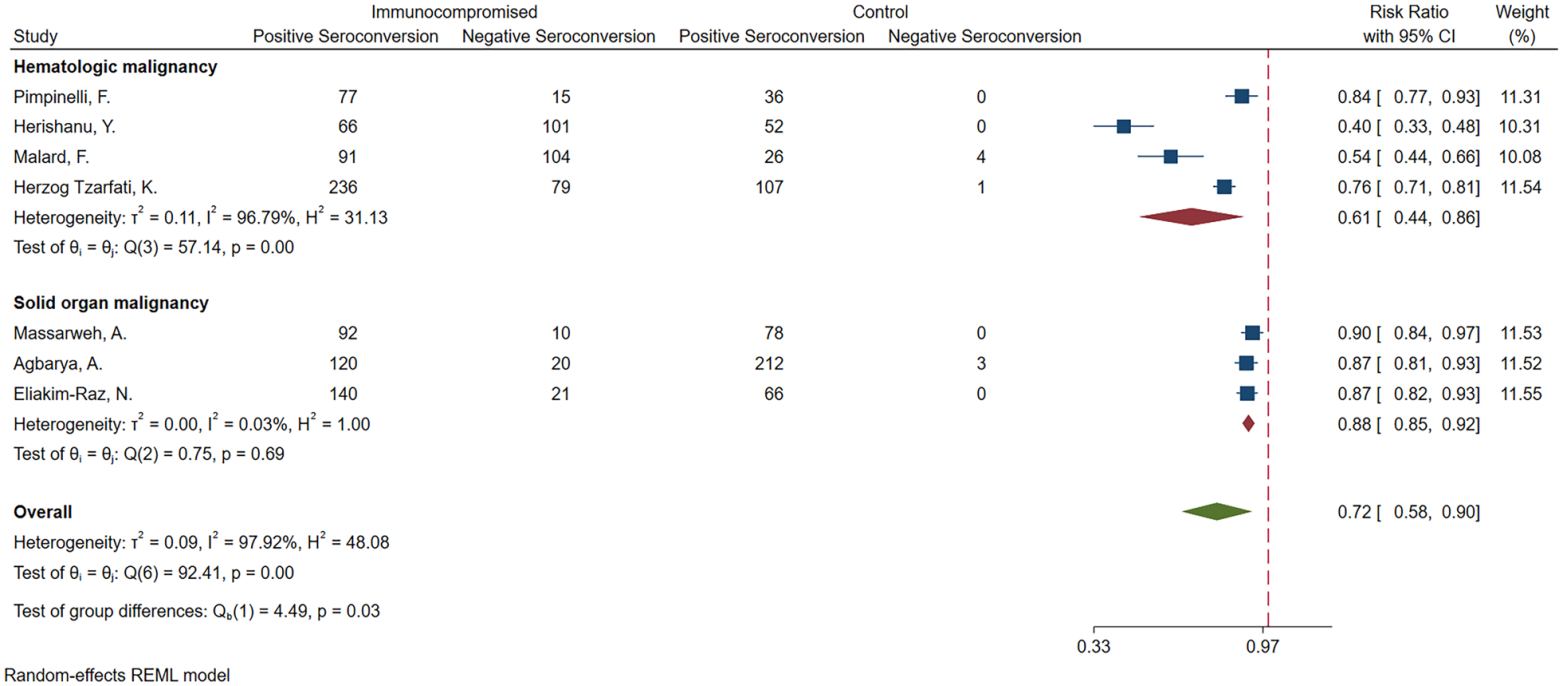
Meta-analysis of seroconversion in immunocompromised patients with malignancy vs controls, based on type of malignancy

Meta-analysis of 9 studies [17, 19, 24, 25, 27, 29, 31, 32, 34] (I^2^ = 97.92) revealed IC patients with malignancy were 0.25 times less likely to seroconvert than healthy controls (RRR = 0.25; 95% CI 0.11, 0.37; *P* < 0.01). Subgroup meta-analysis was conducted based on type of malignancy (hematologic vs. solid organ). Four [24, 25, 29, 34] of the studies were on patients with hematologic malignancy and three [17, 19, 31] were on patients with solid organ malignancy. The relative risk reduction of seroconversion among IC patients with hematologic malignancies was significantly higher than those with solid organ malignancies (RRR = 0.29; 95% CI 0.14, 0.56 vs. RRR = 0.12; 95% CI 0.08, 0.15; *P* = 0.03) (Fig. 3).

### Seroconversion in transplant recipients vs. controls

**Fig. 4:**
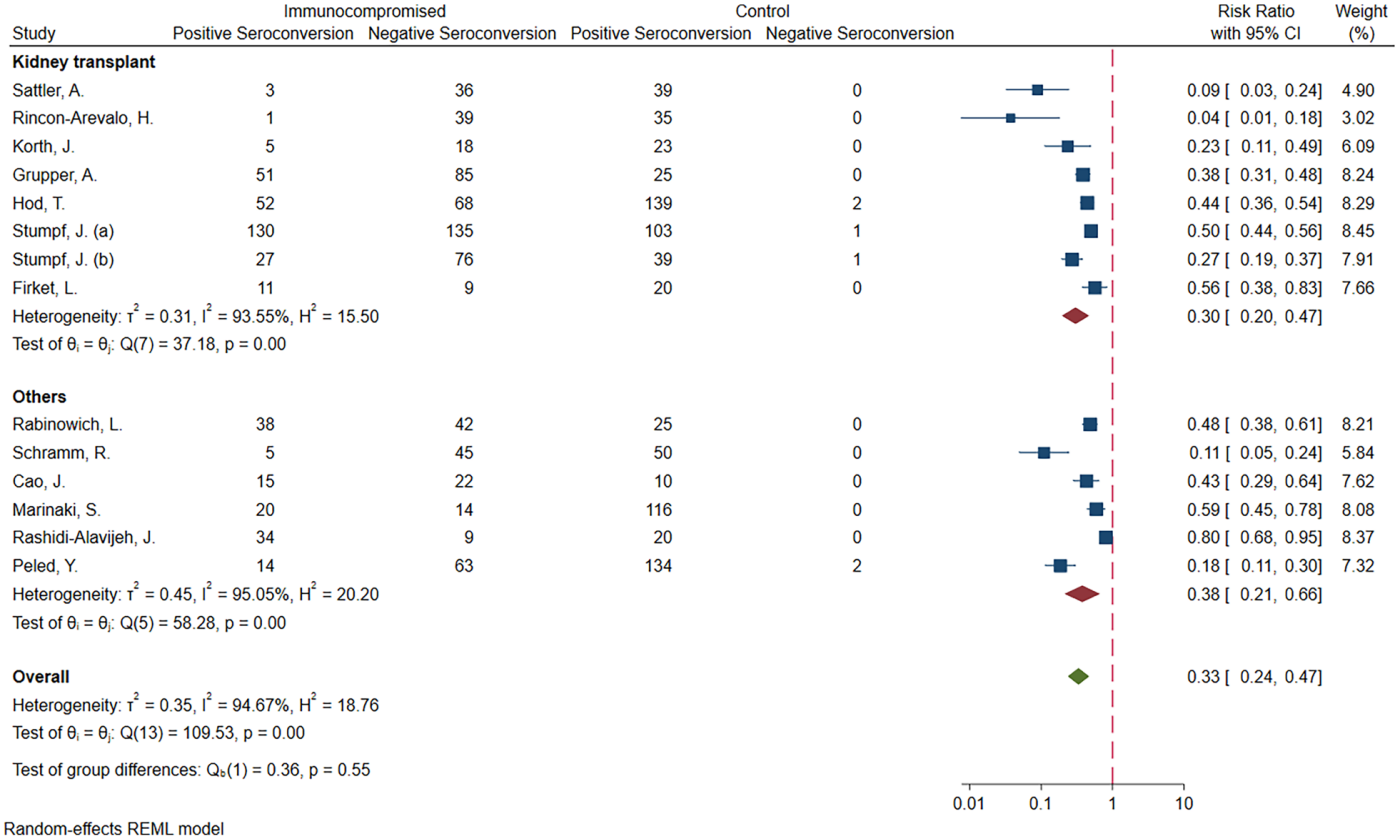
Meta-analysis of seroconversion in transplant patients vs controls, based on type of transplant

Of the included studies, 13 [18, 20, 23, 26, 28, 30, 33, 36, 37, 39–42] were on IC patients due to transplantation (including kidney transplant, heart, lung, and liver). The meta-analysis of the 13 studies (I^2^ = 94.67%) showed transplant recipients were 67% less likely to develop seroconversion than controls (RRR = 0.67; 95% CI 0.53, 0.76; *P* < 0.01). Seven [20, 23, 26, 28, 39, 40, 42] of the included studies were on patients with kidney transplant, and the remaining [18, 30, 33, 36, 37, 41] were on patients with different transplants; none of which with more than three studies to be separated in the subgroup analysis. Hence, a subgroup meta-analysis was conducted based on the type of transplantation (kidney vs. others (including heart, lung, and liver)). The analysis did not reveal any statistically significant difference in relative risk of seroconversion in patients with kidney transplant compared to other types of transplants (RRR = 0.70, 95% CI 0.53, 0.80) vs. RRR = 0.22; 95% CI 0.34, 0.79; *P* = 0.55) (Fig. 4).

### Quality assessment of included studies

Quality assessment of the included studies is presented in Additional file 1: Table S1. The majority of the studies (*n* = 18) [17–19, 22–24, 26, 28, 29, 31, 32, 34, 35, 38–42] were of good quality and 8 [20, 21, 25, 27, 30, 33, 36, 37] had fair quality.

### Publication bias

**Fig. 5:**
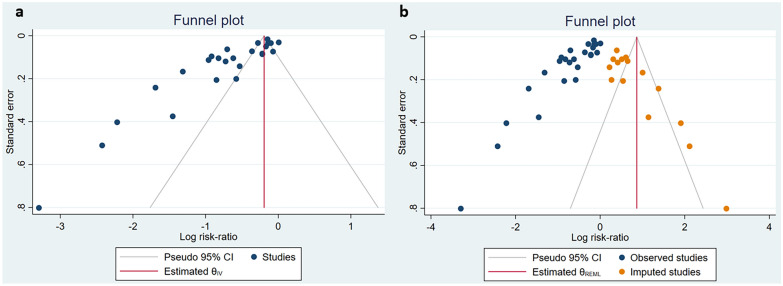
Funnel plot **a** and trim-and-fill funnel plot **b** for meta-analysis of seroconversion in patients with immunodeficiency

Funnel plot for seroconversion was asymmetrical and Egger test showed statistically significant evidence of publication bias (*P* < 0.01, z = − 9.09). Trim-and-fill method was used to adjust the effect size (pooled estimate = 0.87; 95% CI 0.85, 0.88; number of studies = 84) (Fig. 5).

## Discussion

Immunodeficiency comprises a wide range of disorders from primary (e.g., congenital) to numerous secondary conditions acquired consequently to a disease process or its treatment (e.g., human immunodeficiency virus (HIV) infection, radiation therapy, and immunosuppressive medications) [43]. Although inconclusive, it has been shown that IC patients might be at a higher risk of severe COVID-19 [44, 45]. On the other hand, a limited number of studies revealed reduced vaccine efficacy of vaccines in IC patients [46]. Nevertheless, data are limited on the efficacy of COVID-19 vaccines in this critical group of patients.

In this meta-analysis on the immunogenicity of COVID-19 mRNA vaccines in IC patients, we found a lower risk of positive seroconversion in this group of patients compared to healthy controls. In addition, subgroup analysis revealed a significantly lower risk of positive seroconversion in transplant recipients than patients with autoimmune disorder or malignancy. Intriguingly, COVID-19 mRNA vaccines seem to achieve lower efficacy in patients with hematologic malignancies compared to solid organs.

The controls were all healthy individuals, and a lower risk of positive seroconversion might not be surprising as observed with the administration of previous vaccines (e.g., Influenza vaccine) [47]. However, it does not undermine the importance of vaccines in IC patients, as evidence highlights that the immune response after vaccines is more robust than that of natural SARS-CoV-2 infection [48, 49]. It can also imply the importance of booster dose administration in this group of patients. As per recent Center for Disease Control and Prevention (CDC) guidelines, patients with moderately to severely compromised immune systems are recommended to receive an additional dose of COVID-19 mRNA vaccine [50]. Furthermore, studies have shown the promotion of immune response in transplant recipients receiving the third dose of mRNA vaccines, namely mRNA-1273 (Moderna) and BNT162b2 (Pfizer-BioNTech) [51, 52]. However, a dichotomous view toward the booster dose seems insufficient since the degree and etiology of immunosuppression tend to be two important factors regarding immune response and the need for an additional dose [53]. Whether a booster dose is necessarily associated with an enhanced immune response is also a matter of debate. There is evidence that initial post-vaccine antibody titer was predictive of response to booster, and some IC patients will never mount an antibody response [54], and a more restricted personal protection is highly recommended even after vaccination [55].

Interestingly, our analysis revealed significantly lower relative risk of positive seroconversion in patients with transplant compared to patients with autoimmune disorders or malignancies. A study by Evison et al*.* on the efficacy of the Influenza vaccine, showed that the vaccine response rate was higher among patients with HIV and patients who received dialysis compared to renal transplant recipients and patients with a rheumatologic disease [56]. This can be justified by the fact that treatment regimens may be an important contributing factor. Mycophenolate mofetil has been shown to accompany less immune response compared to a regimen consisting of prednisone, cyclosporine, and azathioprine [57–59]. These drugs, which are used to prevent allograft rejection, interfere with T and B cell activation and proliferation, leading to the impediment of antibody generation [60]. Although we did not find any significant difference between kidney transplant and other organ transplant recipients, transplant recipients seem to be more vulnerable to vaccine failures in general, and special attention should be directed toward this group of patients. Studies proposed some approaches to increase the immunogenicity of vaccines in transplant recipients, such as modulation of immunosuppression, adjuvants, intradermal injection, high antigen doses, and booster administration [60].

Hematologic diseases are believed to have the highest level of immunosuppression among malignancies [61]. This group of patients also has 3- to 4-fold higher rates of severe/critical COVID-19 disease and mortality [62, 63]. Hematologic malignancies are associated with immune dysfunction with alterations in both innate and adaptive immunity [64]. Cytopenia, B/plasma cells reduction, hypogammaglobulinemia, and anti-cancer therapy are among the underlying cause of immunodeficiency in these patients [65]; thus, a lower vaccine efficacy might be observed consequently, which is consistent with our findings of the lower immunogenicity of mRNA vaccines in patients with hematologic malignancies.

Although the included four studies demonstrated no statistically significant difference in relative risk of seroconversion between autoimmune disease and control, it still should be interpreted with caution because of limited sample sizes and strong heterogeneity. Autoimmune diseases are a group of heterogenous diseases treated by numerous drugs. For instance, a study of 27 subjects with systemic-onset juvenile idiopathic arthritis (sJIA) found no significant difference between the efficacy of the influenza vaccine in sJIA patients and healthy controls [66]. They also showed that the duration of tocilizumab administration did not impact the response to the vaccine. Also, another recent study showed that although short-term corticosteroid therapy reduces reactogenicity of the first dose of ChAdOx1 nCoV-19, it does not weaken its immunogenicity [67]. On the other hand, a preliminary report (preprint) shows that methotrexate might hamper humoral and cellular immune response to COVID-19 mRNA vaccines [68]. Conspicuously enough, more in-depth investigations are needed in this scope.

It is also worth mentioning that there are numerous approaches to assessing of immune response after vaccine administration which are related to anti-SARS-COV-2 recombinant spike, receptor binding domain, or neutralizing IgG or total antibodies [53]. We included articles with the main outcome of anti-SARS-CoV-2 spike IgG level; however, seropositivity may not necessarily show protection against SARS-CoV-2 [54], and routine assessment of COVID-19 vaccine responses is not recommended [54].

Another important aspect of vaccine immunogenicity can be rendered by T-cell response. T-cell response seems to be achieved efficiently after the second dose of either BNT162b2 mRNA or ChAdOx1 nCoV-19 vaccines [69]. The T-cell response should also be prioritized besides the induction of neutralizing antibodies. T cells are an indispensable part of immune response with the presence of subprotective antibody titers in IC patients [70]; e.g., patients with agammaglobulinemia tend to conquer COVID-19 showing the importance of cellular immune response when there is inefficient humoral response [71–73]. However, there is a lack of data regarding T-cell response in IC patients, and more studies are indeed needed.

We confined this meta-analysis to mRNA vaccines due to limited studies on other COVID-19 vaccine types and to reduce heterogeneity. However, a study by Boekel et al*.* on the development of antibody in patients with autoimmune diseases did not show any significant difference between immunogenicity induced by an mRNA vaccine (BNT162b2) and a viral vector type (ChAdOx1 nCoV-19) [74]. It has also been shown that inactivated COVID-19 vaccine (CoronaVac) can induce an immune response in patients with immune-mediated disease; still, the titer of antibody is associated with age and type of immunosuppressive therapy [75].

This study indeed has some limitations. There was a lack of data regarding HIV and other primary immunodeficiency disorders, and they are not included in this meta-analysis. Furthermore, we included studies with both retrospective and prospective designs, which may reduce the level of evidence. One of the major limitations of this review was the high heterogeneity of the studies. This could be explained by the different quantitative methods used in the studies, different measurement kits and cutoff points to determine a positive seroconversion. Moreover, ethnicity, different types of mRNA vaccines, and study design (i.e., retrospective or prospective) could have been the potential sources of heterogeneity. Further assessment and studies are required in this field.

## Conclusion

The risk of positive seroconversion in IC patients was almost half of those in healthy individuals. However, IC conditions due to autoimmune disorders did not lower the risk of positive seroconversion, but more comprehensive investigations are needed. Among IC conditions, transplant recipients induced the lowest immunogenicity with a 67% lower risk of seroconversion than healthy individuals. Besides, we found that vaccination among IC patients with hematological malignancy induced a lower risk of seroconversion than those among IC patients with solid organ malignancy. Findings from this meta-analysis could aid healthcare policymakers in making decisions regarding the importance of the booster dose or more strict personal protections in the IC patients.

## Supplementary Information

**Additional file 1: Table S1.** Quality assessment using NIH tool.

## Data Availability

The authors stated that all information provided in this article could be shared.

## Abbreviations

IC: Immunocompromised
COVID-19: Coronavirus infectious disease 2019
HIV: Human immunodeficiency virus
