# Supplementary material for: Immunogenicity of COVID-19 mRNA vaccines in immunocompromised patients: a systematic review and meta-analysis
Source: Eur J Med Res. 2022 Feb 12;27:23. doi: 10.1186/s40001-022-00648-5 (PMC8840778; doi:10.1186/s40001-022-00648-5)
Supplement: Supplementary file 1 — Additional file 1: Table S1. Quality assessment using NIH tool. [file 40001_2022_648_MOESM1_ESM.docx]

| **Table S1: Quality assessment using NIH tool** | | |
| --- | --- | --- |
| **Study (First Author)** | **Score (of 14)** | **Quality rating** |
| **Sattler A** | 13 | Good |
| **Rincon-Arevalo H** | 14 | Good |
| **Korth J** | 13 | Good |
| **Rabinowich L** | 9 | Fair |
| **Schramm R** | 11 | Good |
| **Cao J** | 11 | Good |
| **Grupper A** | 11 | Good |
| **Marinaki S** | 9 | Fair |
| **Rashidi-Alavijeh J** | 10 | Fair |
| **Hod T** | 12 | Good |
| **Stumpf J** | 12 | Good |
| **Firket L** | 9 | Fair |
| **Peled Y** | 10 | Fair |
| **Monin L** | 11 | Good |
| **Pimpinelli F** | 12 | Good |
| **Massarweh A** | 11 | Good |
| **Agbarya A** | 11 | Good |
| **Herishanu Y** | 11 | Good |
| **Iacono D** | 10 | Fair |
| **Malard F** | 11 | Good |
| **Eliakim-Raz N** | 11 | Good |
| **Herzog Tzarfati K** | 10 | Fair |
| **Reuken P** | 12 | Good |
| **Geisen UM** | 11 | Good |
| **Furer V** | 10 | Fair |
| **Prendecki M** | 11 | Good |
